# Ambulances are for emergencies: shifting behaviour through a research-informed behaviour change campaign

**DOI:** 10.1186/s12961-019-0517-z

**Published:** 2020-01-23

**Authors:** Kim Borg, David Dumas, Emily Andrew, Karen Smith, Tony Walker, Matthew Haworth, Peter Bragge

**Affiliations:** 1 BehaviourWorks Australia, Monash Sustainable Development Institute, Monash University, 8 Scenic Boulevard, Clayton, Victoria 3800 Australia; 2 The Shannon Company, Melbourne, Victoria Australia; 3 Ambulance Victoria, Doncaster, Victoria Australia; 4 Victorian Department of Health and Human Services, Melbourne, Victoria Australia; 5 Department of Community Emergency Health and Paramedic Practice, Monash University, Frankston, Victoria Australia; 6 Department of Epidemiology and Preventive Medicine, Monash University, Prahran, Victoria Australia

**Keywords:** Ambulance, mass media, behaviour change, emergency service, behavioural intention

## Abstract

**Background:**

A major review of Victoria’s ambulance services identified the need to improve public awareness of the role of ambulances as an emergency service. A communications campaign was developed to address this challenge. This research paper expands on an initial evaluation of the campaign by focusing on the long-term behavioural outcomes.

**Methods:**

The behavioural evaluation involved two types of data collection – administrative data (routine collection from various health services) and survey data (cross-sectional community-wide surveys to measure behavioural intentions).

**Results:**

Behavioural intentions for accessing two of the targeted non-emergency services increased after the second phase of the campaign commenced. There was also a significant change in the slope of call trends for emergency ambulances. This decrease is also likely attributed to the second phase of the campaign as significant level effects were identified 3 and 9 months after it commenced.

**Conclusions:**

A long-term campaign developed through evidence review, stakeholder consultation and behavioural theory was successful in reducing the number of daily calls requesting an emergency ambulance in Victoria and in increasing intentions to use alternative services. This research highlights the importance of collaborative intervention design along with the importance of implementing a robust monitoring and evaluation framework.

## Introduction

Ambulance Victoria (AV), which services Australia’s second most populous state, responds to over 889,000 emergency and non-emergency cases each year [1]. In 2015, it was identified that calls to the ambulance emergency number, 000 (Triple Zero), were increasing faster than the rate of population growth and that many of these calls were not for a genuine medical emergency [2, 3]. The use of limited Triple Zero and AV resources to deal with non-emergency calls reduces the availability of ambulances to rapidly respond to patients experiencing life-threatening emergencies [4]. The 2015 review therefore recommended that public awareness of the role of ambulance services, including when it is appropriate to dial Triple Zero, needed to improve [3].

As part of a suite of strategies in response to this review, the Victorian Government’s Department of Health and Human Services (DHHS) commissioned a public communications campaign aimed at reducing inappropriate calls to Triple Zero for ambulances and increasing the use of other appropriate health services. The program included a formative phase of research, resulting in a decision to run a state-wide campaign. Recognising the importance of robust monitoring and evaluation in this complex area, multiple waves of evaluation research were undertaken to determine the effectiveness of this campaign, respond to any evidence of unintended consequences, and develop additional strategies as required.

### Project objectives

The overall aim of this research was to evaluate the effectiveness of implementing a population-wide, behaviourally informed approach to reducing inappropriate calls for an emergency ambulance in Victoria. The overall evaluation tracked changes in two key areas:

1. Attitudes, awareness and knowledge regarding ambulance use and availability of other services. Results of this analysis have been published elsewhere [5]. In summary, three social surveys (including approximately 1000 participants) – 1 month before and 4 and 10 months after the campaign commenced – revealed that the first phase of the campaign successfully increased community attitudes towards ambulances as being for emergencies only, particularly among those familiar with the campaign material and other non-emergency health services (such as telephone advice lines).
2. Behaviours related to calling Triple Zero for an ambulance – the focus of this paper. Based on previous findings by Borg et al. [5], it was recommended that future iterations of the campaign build on attitudinal messaging by introducing behavioural messaging around what health services Victorians should use for their non-emergency healthcare needs.

This staged approach is underpinned by behaviour change theory. Specifically, the Theory of Planned Behaviour [6] suggests that attitudes are a key predictor of behavioural intentions and, ultimately, behaviours themselves. Based on this theory, we hypothesised that, if the campaign could shift individuals’ beliefs regarding appropriate use of ambulances (i.e. that they are for emergencies only), the community will be less likely to call Triple Zero for an ambulance in a non-emergency once made aware of alternative services.

## Methods

A detailed description of the methods for the formative research and initial evaluation can be found in Borg et al. [5]. The overall approach was based on an established method of exploring and understanding behaviour change challenges and using this information to design and deliver behaviour change interventions [7]. This involved a rapid evidence review (i.e. an overview of reviews), one-on-one consultation interviews with experts in the field, and a structured stakeholder dialogue to deliberate upon findings from evidence and practice. The stakeholder dialogue was attended by 18 people representing the Victorian Government, AV, hospitals, non-emergency health service providers, telecommunications authorities, media strategy, and communications and behavioural researchers. Following deliberation on the pre-circulated information from evidence and practice, it was determined in the dialogue to pursue a long-term behaviour change approach, initially focusing on antecedents to behaviour change.

Following this formative research, the campaign design team (The Shannon Company) generated and tested campaign concepts, resulting in the overall campaign branding of ‘*Save Lives. Save 000 for Emergencies*’. *Will’s Story* was chosen as the most persuasive option for the initial creative design. *Will’s Story* depicted the real-life case of a young boy who required high-level emergency care and helicopter transport to a major metropolitan hospital. The tag line – ‘*it wasn’t luck that saved Will’s life*’ – reinforced the specialist skills of Victoria’s ambulance paramedics and the need to preserve these for life-threatening situations (for a detailed description of *Will’s Story* see Borg et al. [5]). *Will’s Story* ran from March to May 2017 and again from September to November 2017.

Based on the success of *Will’s Story*, it was agreed that the next phase of the *Save 000 for Emergencies* campaign should also be delivered via mass media (TV, print, radio, online, outdoor) but should introduce other health services as options for non-emergency healthcare needs. The rationale for the focus on other healthcare providers was that, if the campaign was trying to discourage a behaviour (dialling Triple Zero for non-emergencies), then an alternative behaviour needed to be provided for those seeking non-emergency healthcare.

The second campaign, entitled *Meet the Team*, featured a nurse from NURSE-ON-CALL,^1^ a pharmacist and a general practitioner (GP). Consistent with the first phase of the campaign:

- The creative content aimed to humanise the health services by presenting likeable, real and relatable individuals who came together as a team;
- The paramedic who featured in *Will’s Story* and the campaign tag line, “*Save Lives. Save 000 for Emergencies*”, were retained for *Meet the Team*, and the closing lines from the healthcare team were “*Save us for emergencies*” (from the paramedic), “*And come to us for everything else*” (from the nurse, pharmacist and GP).

**Fig. 1:**
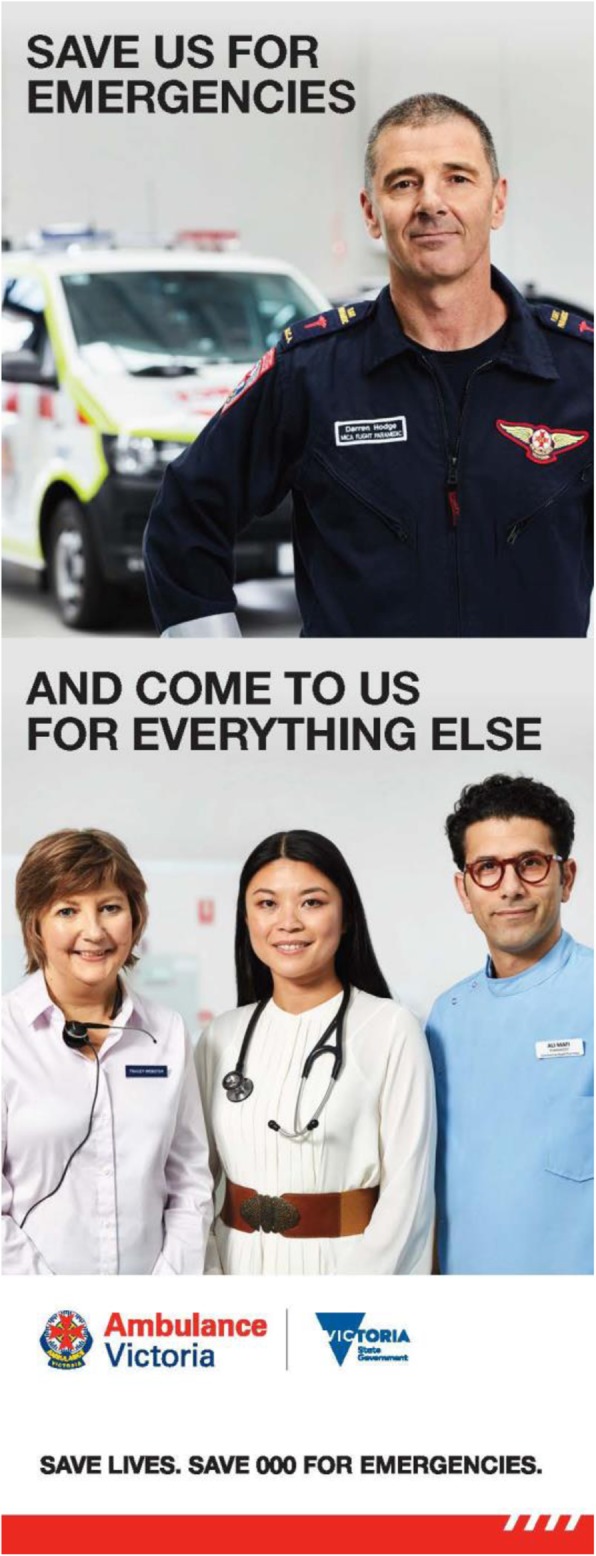
‘Save 000 for Emergencies: Meet the Team’ campaign poster

The healthcare services in *Meet the Team* were presented as complementary options to represent the systemised health support network available in Victoria. Viewers were reminded that, by utilising the network of healthcare options in non-emergencies, they could help save ambulances for emergencies. *Meet the Team* ran from January to April 2018 (Fig. 1).

### Study design

#### Monitoring and evaluation framework

The framework developed to monitor and evaluate the overall campaign programme encompassed four key metrics:

1. Campaign awareness
2. Attitudes towards the use of ambulance services
3. Knowledge of alternative health services
4. Behaviour/use of emergency and non-emergency health services, including behavioural intentions and actual behaviours

Ethics approval to conduct monitoring and evaluation was obtained from Monash University’s Human Research Ethics Committee (#7811). To evaluate the effectiveness of the campaign in changing community-wide behaviours, multiple data sources were included in the evaluation.

#### Administrative data collection

Data on health system usage (for example, calls to Triple Zero for an ambulance, calls to NURSE-ON-CALL and GP attendances) is routinely collected by many agencies for administrative purposes. Administrative data was collated monthly from these sources to track actual behaviours (i.e. usage of and demand for the various services) after the launch of the campaign (March 2017 to October 2018). In addition, data was provided for up to 3 years prior to the campaign (January 2014 to February 2017).

#### Survey data collection

**Fig. 2:**
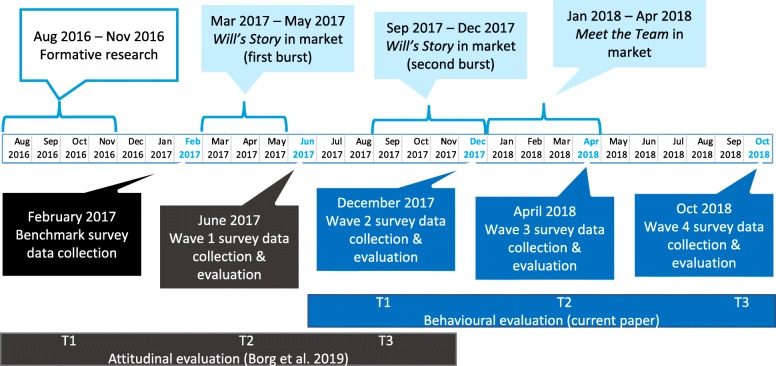
Timeline of campaign and research activities

In total, five community-wide cross-sectional surveys were administered to track attitudes, awareness and knowledge (one prior to and four after launch of the campaign). Questions relating to behavioural intentions were added during the third round of surveying in December 2017, prior to the launch of *Meet the Team*. Therefore, this paper only reports on survey data from December 2017 (hereafter referred to as T1). The first behavioural evaluation survey was conducted in April 2018, 3 months after *Meet the Team* commenced (hereafter referred to as T2), and the final evaluation survey was administered in October 2018, 9 months after *Meet the Team* commenced (hereafter referred to as T3). In order to achieve a representative sample of approximately 1000 respondents per survey round, an online panel research company conducted the surveys with independent cross-sectional samples at each collection period. An overview of the survey data collection rounds as well as other key campaign activities is provided in Fig. 2 below. For more details on the survey procedures see Borg et al. [5].

### Measures

The data used in this study were collected as part of the overall *Save 000 for Emergencies* campaign evaluation. From the larger evaluation, only relevant variables are included in this paper as described below (i.e. those addressing behaviour, rather than attitudes, knowledge and awareness, which have been previously published). These variables relate to metrics associated with (1) behavioural intentions and (2) actual behaviours.

#### Behavioural intentions

Behavioural intentions were measured by asking survey respondents ‘If you needed reliable medical advice or had a health concern that was not an emergency, how likely is it that you would …’ where response options included ‘Call NURSE-ON-CALL’, ‘Visit your local pharmacist’, ‘Visit your local GP’ and ‘Call Triple Zero (000)’. Each response option was then rated on a scale of 0 (very unlikely) to 10 (very likely).

#### Actual behaviour

Data representing calls to Triple Zero requesting an ambulance were provided by AV for the duration of the evaluation period (March 2017 to October 2018). Data was also provided for 3 years prior to the evaluation period (January 2014 to February 2017) for benchmarking purposes. Call data was provided for all Triple Zero requests for an ambulance, ranging from ‘Priority 0’ calls (the highest priority incidents, which require a ‘lights and sirens’ response and additional resources) to ‘Priority 3’ calls (the lowest priority emergency classification). Data was also provided representing calls that were referred to an alternative service or where a non-emergency ambulance was dispatched. In 2016, significant changes were made to the priority classification of Triple Zero calls as part of the suite of strategies implemented in response to the review of Victoria’s ambulance services [3]. Therefore, for the purpose of this evaluation, the total volume of calls was used as an indicator of Triple Zero calling behaviour.

Calls to NURSE-ON-CALL were provided by DHHS and AV – the management of NURSE-ON-CALL moved departments during the evaluation period. Data was provided for the evaluation period (March 2017 to October 2018) and for most of the benchmark period (July 2015 to February 2017); however, data was not available prior to July 2015. GP attendances were tracked using publicly available data from the Australian Government’s Medicare Australia Statistics,^2^ filtered to ‘Professional Attendances – GP Attendances’ for the benchmark period (January 2014 to February 2017) and the campaign period (March 2017 to October 2018). As there is no standard collection method for pharmacy presentations in Victoria, administrative data for pharmacy presentations could not be included in this study.

### Data analysis

All data analysis was conducted using SPSS 23.0 (IBM). Analysis of Variance (ANOVA) were used to assess differences between mean behavioural intention scores across the three survey rounds (T1, T2 and T3). Given the shorter evaluation period for *Meet the Team* (9 months) compared to *Will’s Story* (20 months), ANOVAs were also used to identify the short-term effects of the campaign by comparing the average number of daily calls/visits in the 3-months immediately following the introduction of *Meet the Team* (February–April 2018) compared to the same time in previous years.

Interrupted Time Series Analyses using ARIMA (Auto Regressive Integrated Moving Average) models were used to detect whether or not the campaign had an effect on daily calls/visits during the campaign period [8]. The mean number of calls per day (aggregated) was calculated for each month of available data. Where possible, data was seasonally adjusted to account for changes in health service demand throughout the calendar year. This was done using the Seasonal Decomposition procedure in SPSS, which removes seasonal variation from a series, provided there are at least four full seasons (i.e. 4 years) of data. Analyses were conducted at multiple time points, concurring with the evaluation periods during the course of the programme: June 2017 (4 months after *Will’s Story*), December 2017 (10 months after *Will’s Story*), April 2018 (14 months after *Will’s Story*, 3 months after *Meet the Team*), and October 2018 (20 months after *Will’s* Story, 9 months after *Meet the Team*).

## Results

For each evaluation period, a cross-sectional sample was recruited to broadly reflect the Victorian population regarding age (T1: mean (M) = 45.8 years, SD = 17.4; T2: M = 45.3, SD = 17.2; T3: M = 45.4, SD = 17.3), gender (T1: female = 51.6%; T2: female = 51.3%; T3: female = 51.0%) and location, split between greater capital city (GCC) and the rest of the state (T1: GCC = 75.9%; T2: GCC = 77.3%; T3: GCC = 78.1%).

### Behavioural intentions

Table 1 shows the results of the ANOVA tests for behavioural intentions. The mean likelihood score for ‘Call NURSE-ON-CALL’ increased significantly between T1 (M = 4.42, SD = 3.12) and T3 (M = 4.76, SD = 3.21; *P =* 0.04). Similarly, the mean likelihood score for ‘Visit your local pharmacist’ increased between T1 (M = 5.75, SD = 2.79) and T3 (M = 6.11, SD = 2.69; *P* = 0.01). Likelihood scores for ‘Visit your local GP’ and ‘Call Triple Zero (000)’ did not change significantly between survey rounds. This indicates that people were more likely to intend to seek advice from non-emergency services (nurses and pharmacists) following the launch of *Meet the Team*.

**Table 1:** Likelihood of service use in a non-emergency descriptive statistics

	Mean	SD	95% CI		N	F	*P* value
Call NURSE-ON-CALL							
T1	4.42	3.12	4.23–4.61		1018	2.87	0.04
T2	4.59	3.28	4.39–4.79		1041		
T3	4.76	3.21	4.57–4.96		1034		
Visit your local pharmacist							
T1	5.75	2.79	5.58–5.93		1018	4.38	0.01
T2	5.97	2.78	5.80–6.14		1041		
T3	6.11	2.69	5.95–6.28		1034		
Visit your local GP							
T1	8.07	2.26	7.93–8.21		1018	0.16	0.98
T2	8.13	2.25	7.99–8.27		1041		
T3	8.09	2.23	7.96–8.23		1034		
Call Triple Zero (000)							
T1	1.91	2.80	1.74–2.08		1018	2.00	0.74
T2	1.75	2.79	1.58–1.92		1041		
T3	2.00	2.99	1.82–2.18		1034		

### Actual behaviours

#### Calls to Triple Zero

**Fig. 3:**
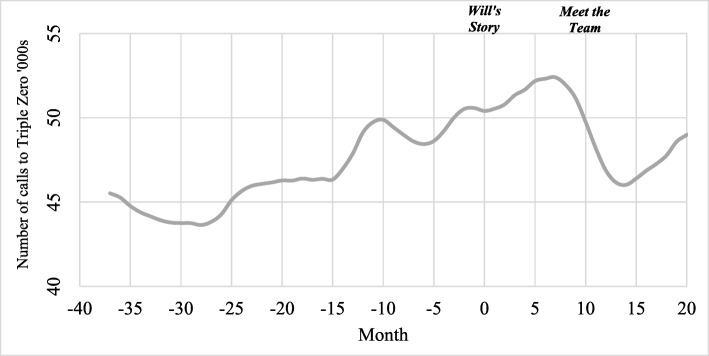
Calls to Triple Zero during benchmark and campaign periods – monthly call volume, seasonally adjusted

Figure 3 presents the total number of calls to Triple Zero each month (adjusted for seasonality using Seasonal Decomposition) during the benchmark period (months −37 to 0; January 2014 to February 2017) and the campaign period (months 1 to 20; March 2017 to October 2018). As seen in Fig. 3, calls to Triple Zero were increasing gradually before any campaign activity commenced. Call volumes continued to increase up to 7 months following the launch of the campaign; however, it is worth noting that this period coincided with a severe influenza season experienced across Victoria in 2017. There was then a comparatively steep decline in daily call volumes between months 8 and 13. *Meet the Team* was in market during months 10 to 14, after which calls started to increase again between months 15 to 20.

Immediately after *Meet the Team* commenced (February to April) ANOVA tests confirmed that there were significant differences each year in mean daily calls to Triple Zero (F = 20.44, df = 2; *P* <0.01). In 2018, there were significantly fewer calls (M = 1474.0, SD = 43.3) compared to the same time period in 2016 (M = 1597.7, SD = 12.6; *P* <0.01) and in 2017 (M = 1619.0, SD: 25.7; *P* <0.01).

Table 2 presents the results of the interrupted time series analyses for Triple Zero call data. Before *Will’s Story* commenced (January 2014 to February 2017), average daily calls to Triple Zero were increasing by 4.8 (*P* = 0.01; 95% CI 1.4 to 8.2). After *Will’s Story* commenced the slope changed to a decrease of 2.9 daily calls (*P* = 0.05; 95% CI − 5.3 to − 0.4). Twenty months after the campaign commenced, there was a decrease of 147.5 daily calls (95% CI − 302.4 to 7.5), which approached but did not achieve statistical significance (*p* = 0.06). Before *Meet the Team* commenced (January 2014 to January 2018), mean daily calls were increasing by 4.6 (*P* <0.01; 95% CI 2.5 to 6.7). The slope of the call trend did not change (*P* = 0.46; 95% CI − 1.4 to 17.7). However, 3 months after *Meet the Team* commenced, daily calls had decreased significantly by 47.8 (*P* <0.01; 95% CI − 76.4 to −19.2), representing a 3.0% relative decrease in average daily calls. A similar finding was noted 9 months after *Meet the Team* commenced (decrease of 47.7 daily calls; *P* <0.01; 95% CI − 76.2 to − 19.2; − 2.9% relative effect).

**Table 2:** Calls to Triple Zero: interrupted time series analysis (seasonally adjusted, average calls per day)

	Phase 1 *Will’s Story*				Phase 2 *Meet the Team*	
	4 months	10 months	14 months	20 months	3 months	9 months
Slope pre-intervention	4.8	4.8	4.8	4.8	4.6	4.6
sig.	0.01	0.01	0.01	0.01	0.00	0.00
Change in slope pre–post	−2.9	−2.9	−2.9	−2.9	8.2	8.2
sig.	0.05	0.05	0.05	0.05	0.46	0.46
Level effect	−24.2	−70.4	− 101.2	−147.5	−47.8	−47.7
sig.	0.28	0.09	0.07	0.06	0.00	0.00
SE	22.0	40.2	54.7	77.3	14.2	14.2
Tinv	44.5	81.1	110.0	155.0	28.6	28.5
CI–	−68.7	− 151.5	−211.2	−302.4	−76.4	−76.2
CI+	20.3	10.7	8.7	7.5	−19.2	−19.2
Predicted value	1679	1674	1523	1593	1527	1600
Relative effect	−1.4%	−4.0%	− 6.2%	−8.5%	−3.0%	−2.9%

While the allocation of priority codes changed during the benchmark period (mid-2016) it was still critical to identify if the campaign influenced the number of urgent calls to Triple Zero. The highest priority code, Priority 0, as well as incidents that were flagged as ‘no treatment, no transport – dead on arrival’ were monitored during the course of the campaign in order to identify potential unintended consequences. Analyses did not identify any significant changes in Priority 0 calls or in ‘dead on arrival’ cases. Further details of these analyses can be found in Appendix.

#### Calls to NURSE-ON-CALL

**Fig. 4:**
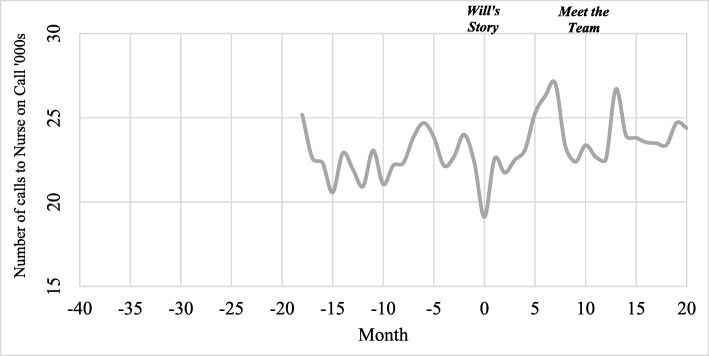
Calls to NURSE-ON-CALL during benchmark and campaign periods – monthly call volume, unadjusted (raw). NURSE-ON-CALL data could not be adjusted for seasonality as data was only available from August 2015. Medial averages for the seasonal factors require at least four full seasons of data

Figure 4 presents the total number of calls to NURSE-ON-CALL each month during the benchmark period (months −18 to 0; August 2015 to February 2017) and the campaign period (months 1 to 20; March 2017 to October 2018). As there were less than 4 years of data, the Seasonal Decomposition procedure could not be conducted, so Fig. 4 represents unadjusted call trends. Call trends to NURSE-ON-CALL fluctuated both before and after the campaign commenced, although there were notable peaks in calls following the launch of *Will’s Story* and *Meet the Team*. It is likely that the increase in calls between months 5 and 7 was also affected by the severe influenza season discussed previously.

ANOVA tests also confirmed that, immediately after *Meet the Team* commenced (February to April), there were significant differences in mean daily calls to NURSE-ON-CALL (F = 14.23, df = 2; *P* <0.01). Specifically, there were significantly more calls in 2018 after *Meet the Team* commenced (M = 821.0, SD = 34.8) compared to the same time in 2016 (M = 722.7, SD = 21.0; *P* = 0.01) and in 2017 (M = 712.0, SD = 25.2; *P <*0.01). This change is likely attributed to *Meet the Team*, given that the phone number for NURSE-ON-CALL accompanied the campaign material. To the best of our knowledge, there were no other factors that could have explained the increase in calls during this time.

Table 3 presents the results of the Interrupted Time Series Analyses for NURSE-ON-CALL data. Before *Will’s Story* commenced, there was no observable trend in average daily calls to NURSE-ON-CALL (*P =* 0.56; 95% CI − 6.3 to 3.4). There were no statistically significant changes in average daily calls after *Will’s Story* started (*P* = 0.36; 95% CI − 5.2 to 8.8). There was also no significant trend in average daily calls before *Meet the Team* commenced (*P* = 0.66; 95% CI − 2.0 to 3.0). Nine months after *Meet the Team* commenced, there was an increase of 63.9 average daily calls, which approached but did not achieve statistical significance (*P* = 0.06; 95% CI − 3.7 to 131.5).

**Table 3:** Calls to NURSE-ON-CALL: interrupted time series analysis (unadjusted, average calls per day)

	Phase 1 *Will’s Story*				Phase 2 *Meet the Team*	
	4 months	10 months	14 months	20 months	3 months	9 months
Slope pre-intervention	−1.4	−1.4	− 1.4	−1.4	0.5	0.5
sig.	0.56	0.56	0.56	0.56	0.66	0.66
Change in slope pre–post	1.8	1.8	1.8	1.8	−4.8	−4.8
sig.	0.36	0.36	0.36	0.36	0.41	0.41
Level effect	42.8	61.9	74.7	93.9	66.5	63.9
sig.	0.25	0.21	0.21	0.23	0.06	0.06
SE	36.4	48.0	58.6	76.4	34.7	33.3
Tinv	76.5	99.0	119.9	155.2	71.1	67.6
CI–	− 33.7	−37.1	−45.2	−61.4	−4.6	−3.7
CI+	119.3	160.9	194.6	249.1	137.6	131.5
Predicted value	749	764	819	828	815	802
Relative effect	6.1%	8.8%	10.0%	8.7%	13.0%	8.7%

#### Visits to GPs

**Fig. 5:**
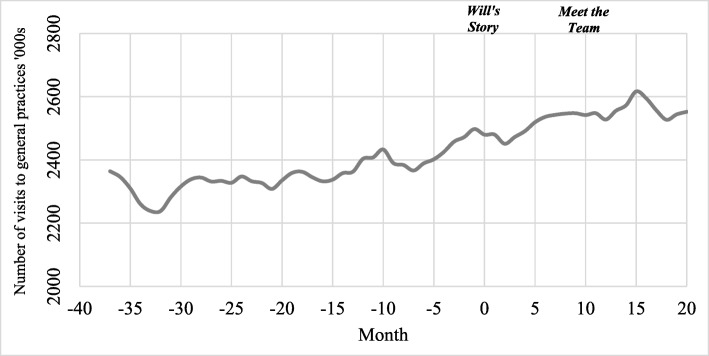
Visits to GPs during benchmark and campaign periods – monthly visit volume, seasonally adjusted

Figure 5 presents the total number of visits to GPs each month (adjusted for seasonality using seasonal decomposition) during the benchmark period (months −37 to 0; January 2014 to February 2017) and the campaign period (months 1 to 20; March 2017 to October 2018). Visits to GPs were trending slightly upwards both before and after the campaign commenced. General practices in Victoria are limited by the number of available spaces to see practitioners and visit clinics; therefore, it is unlikely that any campaign activities would result in a change in attendance trends.

ANOVA tests did not identify any significant differences in visits to GPs (F = 0.193, df = 2; *P* = 0.83) in the 3-month period (February–April) between 2018, 2017 and 2016. This indicates that there were no short-term changes in GP visits after *Meet the Team* commenced.

Table 4 presents the results of the interrupted time series analyses for GP visitation data. Before *Will’s Story* commenced, average daily visits were increasing by 136.9 (*P* = 0.01; 95% CI 42.6 to 231.1). There were no statistically significant changes in visitation trends after the campaign started (*P* = 0.68; 95% CI −59.6 to 436.4). Before *Meet the Team* commenced, average daily visits were increasing by 161.5 (*P* <0.01; 95% CI 87.0 to 235.9). There were no statistically significant changes in visitation trends after *Meet the Team* started (*P* = 0.87; 95% CI − 318.7 to 570.1).

**Table 4:** Visits to general practices: interrupted time series analysis (seasonally adjusted, average calls per day)

	Phase 1 *Will’s Story*				Phase 2 *Meet the Team*	
	4 months	10 months	14 months	20 months	3 months	9 months
Slope pre-intervention	136.9	136.9	136.9	136.9	161.5	161.5
sig.	0.01	0.01	0.01	0.01	0.00	0.00
Change in slope pre–post	188.4	188.4	188.4	188.4	125.7	125.7
sig.	0.68	0.68	0.68	0.68	0.87	0.87
Level effect	253.8	563.4	769.9	1079.5	− 547.7	− 533.3
sig.	0.77	0.68	0.67	0.67	0.47	0.48
SE	870.1	1373.6	1803.1	2493.0	758.2	756.2
Tinv	1763.1	2770.2	3627.4	5000.3	1525.3	1516.7
CI–	− 1509.3	− 2206.7	− 2857.6	− 3920.8	− 2073.1	− 2050.0
CI+	2016.8	3333.6	4397.3	6079.9	977.6	983.4
Predicted value	79,958	82,137	82,485	82,403	82,409	82,245
Relative effect	0.3%	0.7%	0.9%	1.3%	−0.7%	−0.6%

## Discussion

### Summary of findings

The results presented in this paper illustrate that, in addition to improving attitudes towards appropriate ambulance use, as reported previously [5], the *Save 000 for Emergencies* campaign also influenced health-seeking behaviours among the Victorian community. Specifically, there was an increase in intentions to call NURSE-ON-CALL and ‘Visit your local pharmacist’ for non-emergency healthcare needs after *Meet the Team* commenced. The trend in daily Triple Zero calls also changed from increasing before *Will’s Story* commenced to decreasing after it started. We attribute a significant part of this decrease to the launch of *Meet the Team* as level effects were identified 3 and 9 months after it went to market. There was an increase in calls to NURSE-ON-CALL, which coincided with a decrease in calls to Triple Zero during the 3 months after *Meet the Team* commenced, compared to the same time in previous years. However, this short-term change did not translate into a decrease in behavioural trends for any of the services included in the current study. Rather than shifting from Triple Zero to another specific healthcare service, it is likely that those who would have previously called Triple Zero in a non-emergency sought advice or treatment from a number of different healthcare services, essentially dispersing among the Victorian healthcare system.

This study is the most comprehensive known evaluation of a community-wide mass-media behaviour change campaign in this sector. Similar campaign approaches have been adopted in Australia and other countries, but evaluation has been limited. For example, following a campaign about appropriate ambulance use in Japan, the number of ambulance transports was the only measure [9]. Similarly, a previous evaluation of an Australian Triple Zero community awareness campaign assessed ambulance usage, clinical urgency and illness severity of patients attending an emergency department at a hospital in Brisbane [10]. However, neither the Japanese nor the Australian study measured behavioural intentions, attitudes, and campaign awareness.

If Triple Zero calls had been the only measure used in the current evaluation, this may have misled evaluators into thinking the campaign had not been effective in the short-term. However, our results demonstrate that attitude and awareness were precursors of behaviour. Additionally, comprehensive monitoring and evaluation enabled us to identify the unintended consequences of this behaviour change strategy. For example, all emergency ambulance services experience a small number of cases where patients should have called for an ambulance but did not. It was therefore critical to identify if the campaign reduced the number of time-critical, life-threatening emergency calls. Our results indicate that this campaign did not influence the number of calls in the highest priority emergency category.

Limitations of this project include a lack of a control group; while the study design was appropriate for evaluating the effectiveness of a mass media campaign, we cannot be certain that the changes observed in the study were the direct result of the campaign (attribution). For example, the severe influenza season in Victoria in 2017 may explain the increase in calls to Triple Zero and NURSE-ON-CALL during this period. There were 12,348 laboratory-confirmed cases of influenza in Victoria by the start of September 2017 [11] – more than double the comparable period in 2016 (6666) [12]. This likely led to an increase in both emergency and non-emergency health-seeking behaviours like influenza vaccinations [13].

A further limitation is the limited consistency and availability of some administrative data, which meant that certain analyses could not be performed uniformly across data sets. For example, NURSE-ON-CALL did not have enough benchmark data available for seasonal adjustments. Similarly, due to significant changes to the Triple Zero dispatch model in 2016 (where a large number of calls were redirected to non-emergency services), it was not possible to consistently identify non-emergency calls to Triple Zero throughout the benchmark and campaign periods. Finally, the lack of standard data collection for pharmacy presentations in Victoria meant that administrative data for pharmacies, which were promoted in *Meet the Team*, could not be included in this study.

The final limitation relates to the behavioural intentions survey measure. Respondents were asked about their likelihood of engaging in a series of behaviours specifically in a non-emergency situation, which could be interpreted as a leading question where calling the Triple Zero emergency service is assumed to be an erroneous response. The question also did not capture behavioural intentions, which are the result of genuine misconceptions about what constitutes an emergency healthcare need. While we recognise these limitations, we also note that designing a measure of the behavioural intentions in a non-emergency situation is quite complex. As found in the formative research phase of this research, definitions of ‘emergency’ and ‘non-emergency’ are not definitive in research literature or practice, and even members of the medical field often have contrasting perspectives on what constitutes a medical emergency [5]. Therefore, specific symptoms were avoided throughout the entire campaign and evaluation so that the focus was on individual’s responses to situations that they perceived to be a non-emergency (e.g. the primary attitude measures from the initial evaluation were ‘Ambulances are for All’ and ‘Ambulances are for Emergencies’).

### Theoretical, practical and policy implications

The findings of this project are consistent with the Theory of Planned Behaviour, which posits that behavioural intentions are influenced by attitudes (toward the behaviour), subjective norms (perceived social pressure to engage in the behaviour) and perceived behavioural control (apparent ease or difficulty of engaging in the behaviour) [6]. Mass media campaigns can ostensibly create a sense of social pressure because the messages are delivered on a large-scale and in a public manner so audiences not only receive the message but they know that the message is being received by many others [14]. The *Save 000 for Emergencies* campaign further harnessed the Theory of Planned Behaviour by first addressing attitudes towards the behaviour via *Will’s Story.* This promoted Victorian ambulance paramedics as a highly skilled workforce for saving lives, which should be reserved for life-threatening emergencies. The campaign then maintained this attitudinal message while adding a perceived behavioural control message via *Meet the Team*, which introduced alternative and easily accessible services for non-emergency conditions (GPs, pharmacists and NURSE-ON-CALL).

The findings from this research demonstrate the merits of sensitising the market (*Will’s Story*) before moving to a specific call to action (*Meet the Team*). They are also consistent with results of other major behaviour change campaigns (e.g. smoking cessation, workplace safety and safe driving [15–17]), which indicate that continued exposure to carefully constructed mass media campaigns can play an integral role in population-level behaviour change. To sustain this change, like these previous campaigns, ongoing exposure to the campaign message should be considered.

We cannot overstate the importance of multi-sector engagement in the planning and execution of this project – that is, bringing together AV, DHHS, relevant telecommunications authorities, media strategy and communications organisations, and behavioural researchers. For example, a key lesson from the formative research was that any attempt to define an emergency should be avoided for two reasons. First, there are hundreds of emergency response codes and conditions that could not be comprehensively communicated in a public campaign. Second, attempts to classify emergencies into simplified categories, for example, red (emergency), orange (urgent and potential emergency) and green (non-emergency) are fraught and potentially dangerous because symptoms can be indicative of a range of conditions that could be classified across different categories. For example, a headache could be mild dehydration (orange) or it could be symptomatic of a stroke (red); back pain could be a pulled muscle (green) or a sign of an abdominal aortic aneurysm (red). Such ambiguity was considered too risky in the context of this Triple Zero campaign.

Collectively, the formative research, multiple phases of campaign development and ongoing evaluation required considerable investment. However, from the perspective of the project partners (particularly AV and DHHS) such an investment was considered worthwhile to manage service demand. This underscores the challenge of shifting behaviour at a community level and reinforces the need to secure a shared understanding between collaborating and funding partners of the complexity of the task and the time taken to yield results. Specifically, in deciding to undertake a state-wide public campaign, it was important to emphasise to policy-makers within DHHS that such an approach would not likely change Triple Zero calls in the short term, as attitudes needed to shift in advance of behaviour.

It is also important to consider the unintended consequences of not using behavioural theory to guide public health campaigns. For example, we know from both behavioural literature and the experiences of other state-based campaigns in Australia, that advertisements that tell viewers what not to do can unintentionally promote the highlighted behaviour through social norming. That is, once people become aware that Triple Zero has been used by others for trivial health matters, calls to Triple Zero can actually increase. Such messaging was specifically avoided for this reason, instead focusing on what to do – save ambulances for emergencies.

From a policy and practice perspective, the results of the evaluations illustrate the utility of taking an evidence-based approach to designing a state-wide campaign, along with implementing a robust monitoring and evaluation framework. In an era of rapid policy response demands, driven by a desire for instant results, this research demonstrates how a holistic model of campaign development can effectively contribute to changing such an ingrained community behaviour as calling Triple Zero for an ambulance. Based on the outcomes of this project, consideration will be given to applying this model to future applicable behaviour change campaigns within DHHS.

### Future directions

For continuity and in order to build cumulative awareness, any future campaign executions should continue to use existing campaign assets, such as the four members of the healthcare team and the campaign tagline, ‘*Save lives. Save 000 for Emergencies*’. The next phase of the campaign could also provide more detail on specific services provided by alternative health services; for example, the ability of pharmacists in Victoria to treat, advise and refer for certain conditions. Ongoing monitoring and evaluation of any future iterations of the campaign will be critical to further building understanding of the ongoing exposure effect of variations in campaign intensity on community-level behaviour. Ongoing evaluations could also examine whether cessation of the campaign is associated with the volume of Triple Zero calls returning to previous trends – noting that other public health campaigns, such as road safety, have been in place in some form for decades [18].

## Conclusions

A long-term public campaign, *Save 000 for Emergencies*, developed through evidence review and stakeholder consultation and based on behavioural theory, was successful in reducing the number of calls for an emergency ambulance in Victoria and in increasing intentions to use alternative, non-emergency services (NURSE-ON-CALL and pharmacies). The resource intensity and time taken to develop and execute this campaign reflects the challenge of shifting behaviour at a whole-of-population level and mirrors the long-term investment in similar public health campaigns such as those on road safety and cigarette smoking. Critical to the success of the campaign was a deep understanding of behaviour change theory and close collaboration with government, emergency services, telecommunications authorities, providers of non-emergency health services, communications specialists and academics. Comprehensive monitoring and evaluation enabled measurement of both target outcomes and unintended consequences. Further monitoring and evaluation can establish the effect of variations in campaign intensity (including cessation of the campaign) on the target attitudes and behaviours.

## Data Availability

De-identified summary data that support the findings of this study are available from the corresponding author on request and subject to the approval from the Victorian Department of Health and Human Services and the original owners of the data.

## Appendix

### Unintended consequence analyses

**Table 5:** Calls to Triple Zero: interrupted time series analysis (seasonally adjusted, average calls per day) – Priority 0 and DOA^a^ cases

	Phase 2 *Meet the Team* (9 months)			
	Priority 0	DOA	Priority 0	DOA
Slope pre-intervention	0.0	0.0	0.0	0.0
sig.	0.93	0.71	0.67	0.17
Change in slope pre–post	0.0	0.1	−0.1	0.0
sig.	0.90	0.49	0.56	0.70
Level effect	−0.9	1.1	−1.1	−0.4
sig.	0.87	0.58	0.19	0.46
SE	5.6	2.0	0.8	0.5
Tinv	11.3	4.0	1.7	0.9
CI–	−12.2	−2.9	−2.8	− 1.3
CI+	10.3	5.1	0.6	0.6
Predicted value	39	9	39	9
Relative effect	−2.4%	14.3%	−2.8%	−3.7%

^a^Note: Priority 0 are the highest priority incidents which require a ‘lights and sirens’ response and usually involve sending additional resources such as a Mobile Intensive Care Ambulance. DOA are cases flagged as ‘not treated and not transported’ with the reason as ‘dead on arrival’

## Abbreviations

AV: Ambulance Victoria
DHHS: Department of Health and Human Services
GP: General practitioner

## Footnotes

1 *NURSE-ON-CALL* is a phone service that provides immediate, expert health advice from a registered nurse, 24 h a day, 7 days a week.

2 http://medicarestatistics.humanservices.gov.au/statistics/mbs_group.jsp
